# Supplementary material for: Nonspecific membrane bilayer perturbations by ivermectin underlie SARS-CoV-2 in vitro activity
Source: bioRxiv. 2023 Oct 24:2023.10.23.563088. Preprint. [Version 1] doi: 10.1101/2023.10.23.563088 (PMC10634736; doi:10.1101/2023.10.23.563088)
Supplement: Supplement 1 [file media-1.pdf]

Nonspecific membrane bilayer perturbations by ivermectin underlie SARS-CoV-2 *in vitro* activity

Richard T. Eastman[#], Radda Rusinova[#], Karl F. Herold[#], Xi-Ping Huang[#], Patricia Dranchak, Ty C. Voss, Sandeep Rana, Jonathan H. Shrimp, Alex D. White, Hugh C. Hemmings Jr., Bryan L. Roth, James Inglese, Olaf S. Andersen[*], Jayme L. Dahlin[*]

Table of contents

Supplementary figures

S1. Cellular health control compounds S2
S2. Cellular health ivermectin analogs S3
S3. Complete media content can modulate effect of ivermectin on cell viability S4
S4. Alpha technology interference profiles of reference compounds S5
S5. Ivermectin is unlikely to interfere with biological assays by colloidal aggregation or drug-induced phospholipidosis S7
S6. Ivermectin and chemical analogs do not inhibit several SARS-CoV-2-related targets in cell-free assays S7
S7. Ivermectin does not inhibit recombinant luciferases S8
S8. Quality control of primary ivermectin sample S9

Supplementary tables

S1. Summary of ivermectin target activities S10
S2. Activity summary of ivermectin samples in PubChem Alpha-based assays S11
S3. Normalized fluorescence quench rates of ivermectin analogs S14
S4. Compound quality control and source summary S15

Supplementary notes

Note 1. Antiparasitic structure-activity relationships for ivermectin and analogs S16
Note 2. Ivermectin is not an effective SARS-CoV-2 antiviral in high-quality clinical trials S17

Detailed author contributions S18

Supplementary references S19

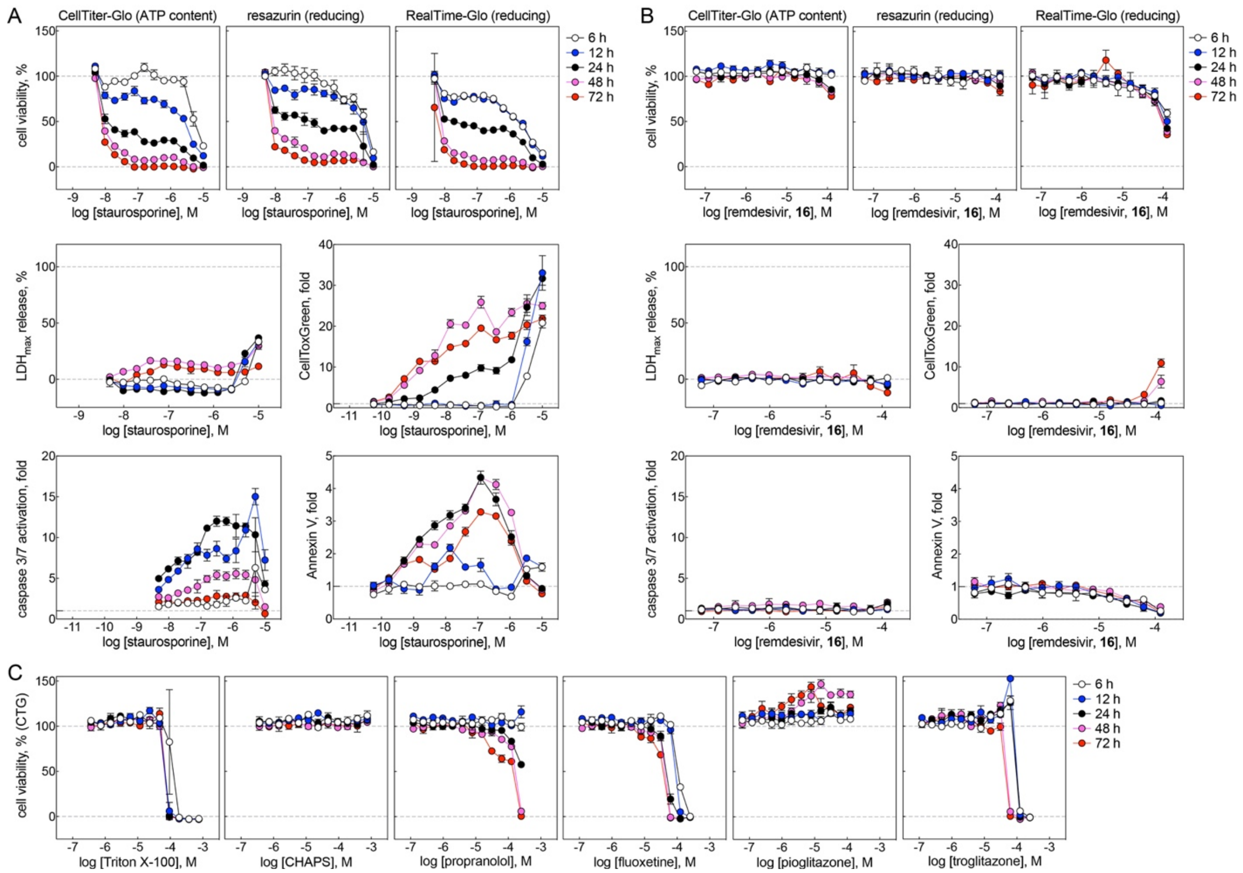

Supplementary Figure 1. Cellular health control compounds. (A) Positive cytotoxicity control compound staurosporine decreases cellular viability (CTG, resazurin, RTG), causes A549cytotoxicity (LDH, CellToxGreen), and increases biochemical markers of apoptosis (caspase 3/7, Annexin V) at nanomolar compound concentrations in A549-ACE2 cells. Data are mean ± SD of four intra-plate technical replicates. (B) Control compound remdesivir only decreases cellular viability, causes cytotoxicity, and increases biochemical markers of apoptosis at high micromolar concentrations in A549-ACE2 cells. Data are mean ± SD of four intra-plate technical replicates. (C) Select membrane bilayer modifying control compounds decrease A549-ACE2 viability at micromolar concentrations. Triton X-100, propranolol, fluoxetine, troglitazone: positive cytotoxic controls; CHAPS, pioglitazone: negative cytotoxic controls. Data are mean ± SD of four intra-plate technical replicates. Experiments were performed in parallel with **Figure 2**. Source data are provided as a Source Data file.

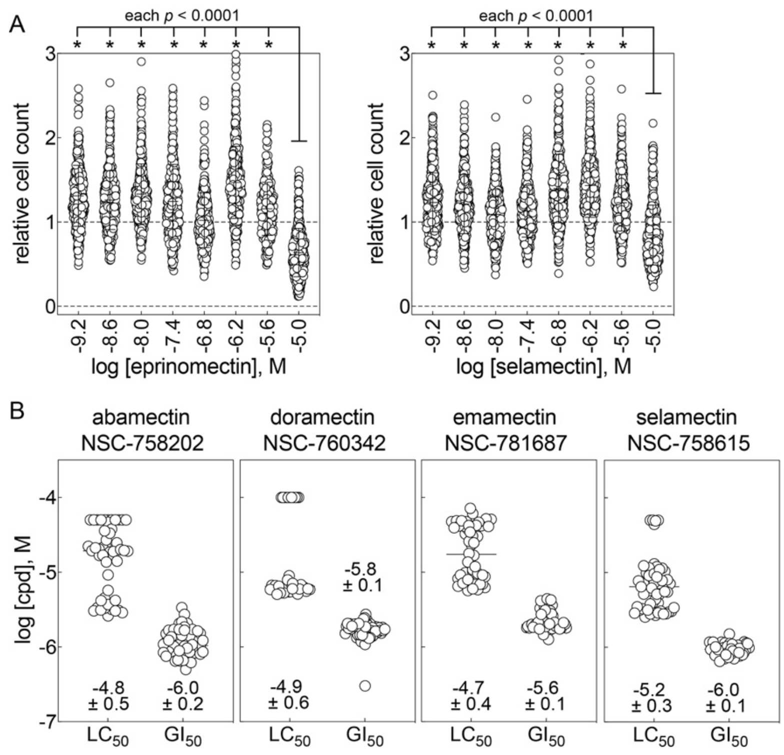

Supplementary Figure 2. Cellular health ivermectin analogs. (A) The IVM analogs eprinomectin and selamectin broadly decrease cell counts at low micromolar concentrations in the Broad PRISM assay (Broad DepMap, https://depmap.org/portal/). Each data point represents a unique cancer cell line. Statistical differences determined by ordinary one-way ANOVA adjusted for multiple comparisons. (B) The IVM analogs abamectin, doramectin, emamectin, and selamectin broadly decrease observed cell counts and inhibit cell growth at low micromolar concentrations in the NCI60 assay (https://dtp.cancer.gov). Each data point represents a unique cancer cell line. LC_{50}, drug concentration resulting in a 50% reduction in the measured protein at the end of the drug treatment as compared to that at the beginning; GI_{50}, drug concentration resulting in a 50% reduction in the net protein increase as measured by sulforhodamine B staining in control cells during the drug incubation. Source data are provided as a Source Data file.

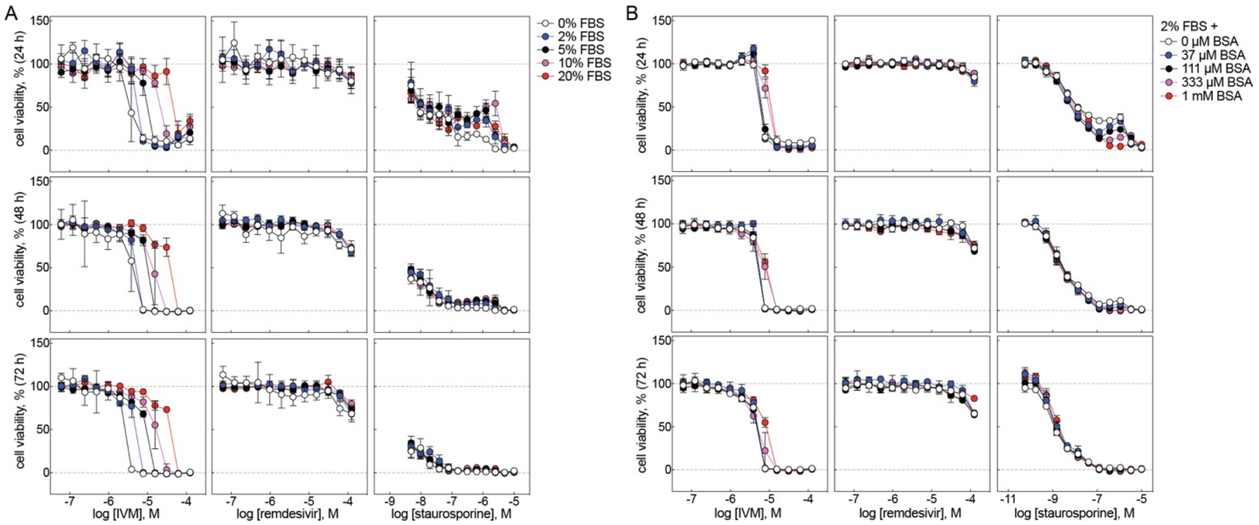

Supplementary Figure 3. Complete media content can modulate effect of ivermectin on cell viability. (A) The effect of IVM on A549-ACE2 cell viability depends on the FBS concentration in complete media. This effect is not observed for remdesivir or staurosporine. Data are mean ± SD of four intra-plate technical replicates. (B) The effects of IVM, remdesivir, and staurosporine on A549-ACE2 cell viability does not grossly depend on the BSA concentration in complete media. Note that 2% FBS contains approximately 7.5 μM final albumin concentration, based on an expected albumin concentration of 2.5 g/dL in 100% FBS. Data are mean ± SD of four intra-plate technical replicates. Source data are provided as a Source Data file.

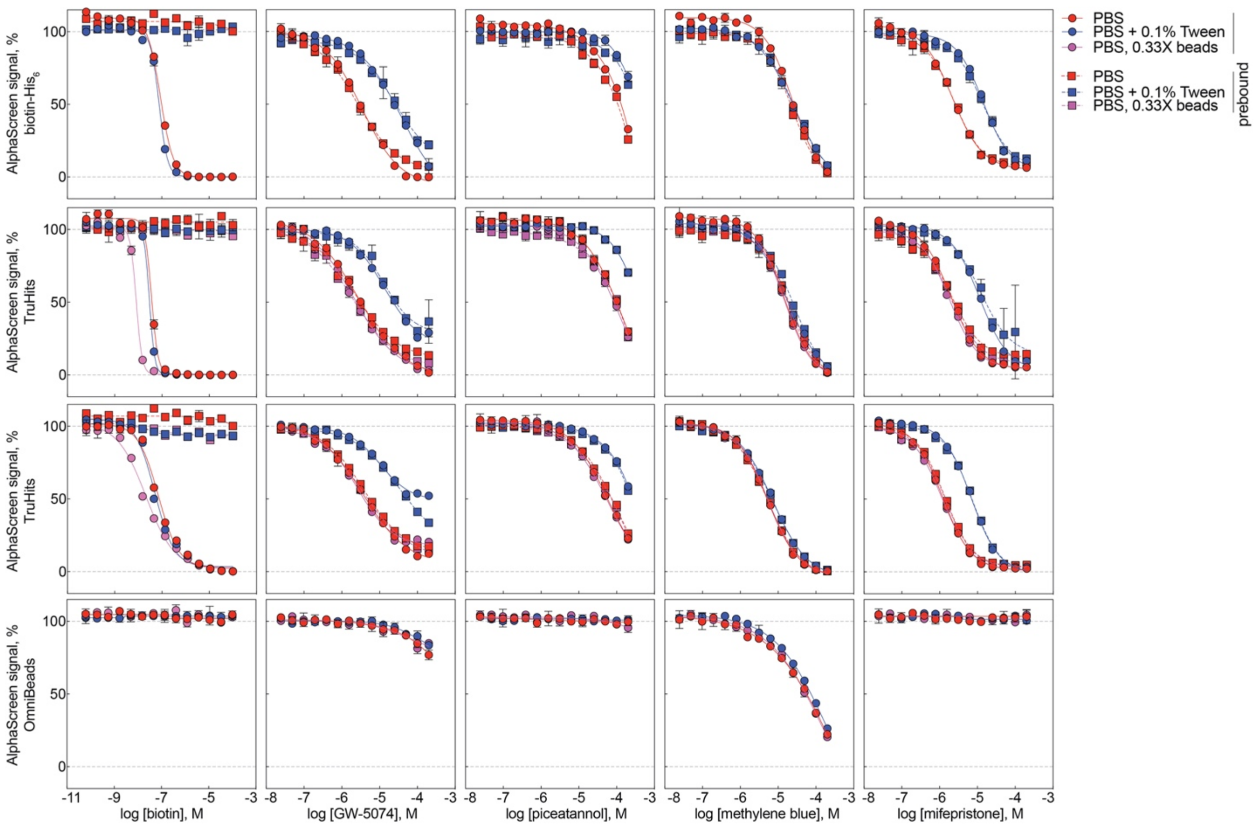

Supplementary Figure 4. Alpha technology interference profiles of reference compounds. Compound-mediated interference with the Alpha homogenous proximity technology was assessed according to the NCATS *Assay Guidance Manual*.[1] Biotin, prototypical capture reagent disruptor (streptavidin-biotin interaction); GW-5074, prototypical light scatterer; piceatannol, singlet oxygen quencher; methylene blue, prototypical colored/light absorbance; mifepristone, reported NLS-importin inhibitor.[2] Data are mean ± SD of four intra-plate technical replicates. Source data are provided as a Source Data file.

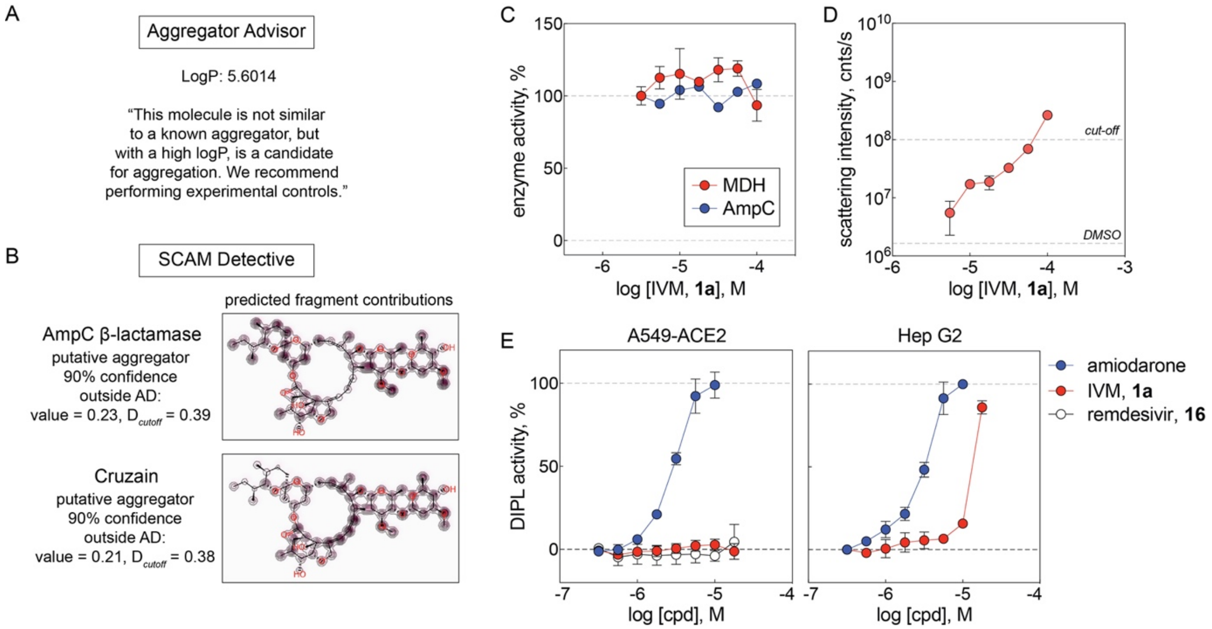

Supplementary Figure 5. Ivermectin is unlikely to interfere with biological assays by colloidal aggregation or drug-induced phospholipidosis. (A) IVM is predicted to be a candidate colloidal aggregator by the Aggregator Advisor tool due to its high ClogP (https://advisor.bkslab.org; accessed 07 Oct 2023).[3] Shown is the analysis output. (B) IVM is predicted to be a putative aggregator with high confidence by the SCAM Detective tool (https://scamdetective.mml.unc.edu/; accessed 07 Oct 2023).[4] Shown are the analysis outputs from its models based on AmpC β-lactamase and cruzain, although the structure was flagged as outside the applicability domain. (C) IVM does not inhibit the aggregator-sensitive enzymes AmpC and MDH in the absence of detergent. MDH: data are mean ± SD of three intra-plate technical replicates; AmpC: data are a single point. (D) IVM does not form colloids at low micromolar concentrations as detected by DLS. Data are mean ± SD of three intra-plate technical replicates. (E) IVM does not induce DIPL in A549-ACE2 cells, but induces DIPL at high micromolar concentrations in Hep G2 cells. Amiodarone, positive DIPL control; remdesivir, negative control. Data are mean ± SD of three independent experiments. Amiodarone, positive DIPL control; remdesivir, negative control. Source data are provided as a Source Data file.

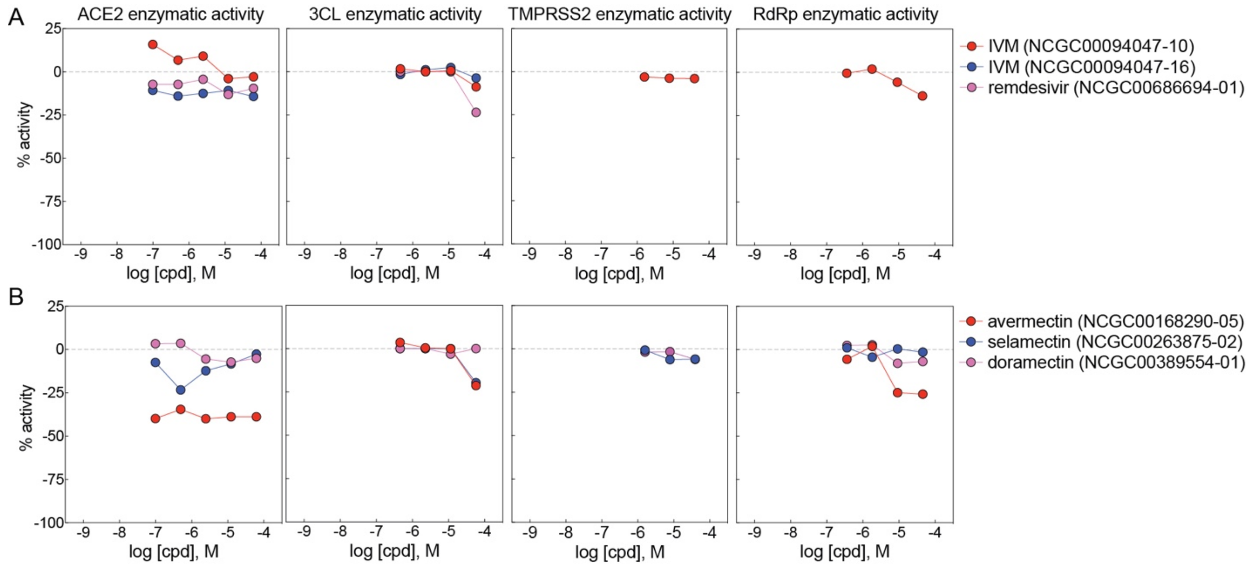

Supplementary Figure 6. Ivermectin and chemical analogs do not inhibit several SARS-CoV-2-related targets in cell-free assays. (A) Two IVM samples do not inhibit the SARS-CoV-2 targets 3CL or RdRp, as well as human ACE2 and TMPRSS2, in biochemical assays. Data are single points from an inter-plate qHTS. (B) The IVM analogs avermectin, selamectin, and doramectin do not inhibit the SARS-CoV-2 targets 3CL or RdRp, as well as human ACE2 and TMPRSS2, in biochemical assays. Data are single points from an inter-plate qHTS. Data is from NCATS OpenData Portal. NCGC numbers indicate specific samples. Source data are provided as a Source Data file.

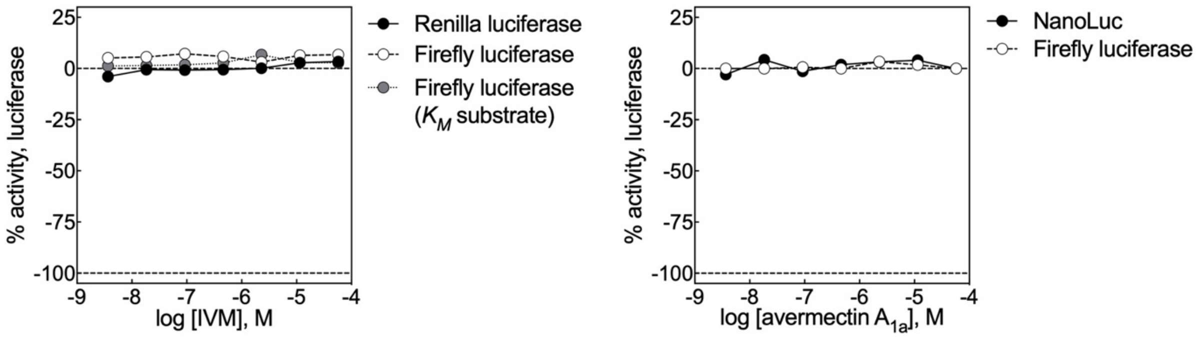

Supplementary Figure 7. Ivermectin does not inhibit recombinant luciferases. Data expressed as percent change relative to DMSO control, with 0% being no effect, and -100% being complete inhibition. Data are single points from quantitative high-throughput screening.[5] Source data are provided as a Source Data file.

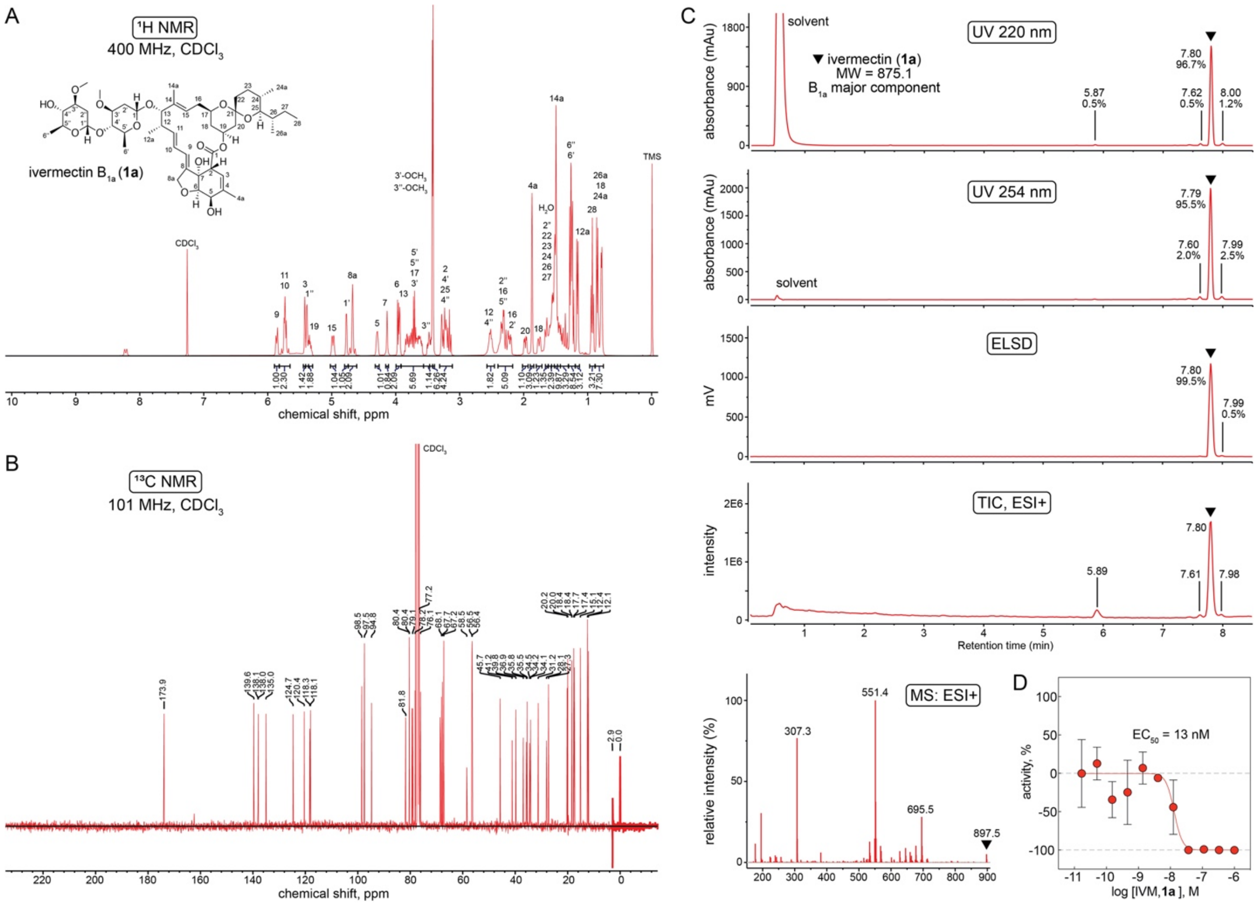

Supplementary Figure 8. Quality control of primary ivermectin sample. The primary study sample of IVM in this study (**1a**) was obtained as a USP standard. (A) ^{1}H NMR of primary IVM sample is consistent with its reported chemical structure.[6] (B) ^{13}C NMR of primary IVM sample (**1a**) is consistent with its reported chemical structure. (C) Primary IVM sample shows acceptable purity by UPLC-MS, as quantified by UV220 nm, UV254 nm, and ELSD. The parent ion as detected by UPLC-MS (ESI, + mode) is consistent with its reported chemical structure. (D) Primary IVM sample (**1a**) shows expected anthelmintic activity at nanomolar concentrations versus *Caenorhabditis elegans*. Data are mean ± SD of four inter-plate biological replicates. N.b., data included as part of **Figure 1C**. Source data are provided as a Source Data file.

Supplementary Table 1. Summary of ivermectin target activities.

target	cys-loop receptor	concentration (range)	reference
GluCl	yes	100 nM	[7]
histamine-gated chloride channel	yes	1 μM	[8]
H^+-gated chloride channel	yes	10 μM	[9]
GlyR	yes	1 μM	[10]
$GABA_A$	yes	10 μM	[11]
α7-nAChR	yes	30 μM	[12]
RYR	no	1 μM	[13]
SERCA	no	10 μM	[13]
Na,K-ATPase, H,K-ATPase	no	10 μM	[14]
P2X4	no	1 μM	[15]
hERG	no	10 μM	[16*]
GIRK	no	10 μM	[17]
various xenobiotic transporters	no	1 – 10 μM	[18]

*, may reflect decreased trafficking[19]

Supplementary Table 2. Activity summary of ivermectin samples in PubChem Alpha-based assays. Relevant PubChem biological assays for ivermectin using Alpha-based technology were identified by the following searches: "alphascreen[All Fields] ivermectin", "alphalisa[All Fields] ivermectin", and manual inspection of bioassay records from PubChem CIDs 3085416, 6321424, 11957587, 45114068, and 73265241. Assay activity and protocol information were then extracted by manual review of PubChem assay descriptions and protocols. All assays used AlphaScreen format unless noted. Data accessed 21 July 2023 (https://pubchem.ncbi.nlm.nih.gov/).

PubChem AID	PubChem CID	PubChem SID	assay name	compound concentration (μM)	activity score	activity summary	detergent	notes
1259374	11957587	321942436	AlphaScreen-based biochemical high throughput primary assay to identify inhibitors of microphthalmia-associated transcription factor (MITF)	2.6	-3.95%	inactive	0.1% Tween-20	
		332950534		2.6	-33.63%	inactive		
		332950174		2.6	-19.12%	inactive		
	45114068	333494472		2.6	-3.99%	inactive		
		333494081		2.6	7.98%	inactive		
1259310	11957587	321942436	AlphaScreen-based biochemical high throughput primary assay to identify activators of the E3 ligase (FBW7)	26.1	0%	inactive	0.1% Tween-20	
		332950534		26.1	-0.04%	inactive		
		332950174		26.1	0.43%	inactive		
	45114068	333494472		26.1	0.05%	inactive		
		333494081		26.1	0.12%	inactive		
1454	11957587	50106505	qHTS assay for inhibitors of the ERK signaling pathway using a homogeneous screening assay; stimulation with EGF	0.460<br>11.49	-4.79%<br>3.37%	inconclusive	information not provided	lytic cellular assay; lysis buffer may contain detergent
485290	11957587	50106505	qHTS assay for inhibitors of tyrosyl-DNA phosphodiesterase (TDP1)	0.004<br>0.021<br>0.094<br>0.505<br>2.283<br>12.29<br>56.06	13.46%<br>4.41%<br>4.11%<br>3.88%<br>3.96%<br>8.38%<br>13.46%	inactive	0.05% Tween-20	
1477	11957587	50106505	qHTS assay for compounds blocking the interaction between CBF-beta and RUNX1 for the treatment of acute myeloid leukemia	0.093<br>0.514<br>2.359<br>13.09<br>59.83	2.28%<br>5.12%<br>7.18%<br>5.67%<br>0.61%	inactive	0.01% Tween-20	
504332	11957587	50106505	qHTS assay for inhibitors of histone lysine methyltransferase G9a	0.018<br>0.092<br>0.501<br>2.335<br>12.70	20.01%<br>33.54%<br>32.02%<br>20.37%<br>21.19%	inactive	information not provided	
	73265241	90340581		0.004<br>0.018<br>0.092<br>0.501<br>2.335<br>12.70<br>58.95	-4.98%<br>-6.78%<br>-3.27%<br>-4.71%<br>48.57%<br>33.37%<br>26.62%	inactive		

1259251	11957587	321942436	AlphaScreen-based biochemical high throughput primary assay to identify inhibitors of Unc-51 like autophagy activating kinase 1 (ULK1)	5.96	-0.03%	inactive	0.01% Triton X-100	
488949	11957587	50106505	qHTS validation assay for inhibitors for MPP8 chromodomain interactions with methylated histone tails	0.018<br>0.091<br>0.457<br>2.290<br>11.40<br>57.10<br>114.0	13.16%<br>-3.34%<br>-3.19%<br>12.13%<br>4.03%<br>-40.23%<br>-39.25%	inconclusive	0.01% Tween-20	
	73265241	90340581		0.004<br>0.018<br>0.091<br>0.457<br>2.290<br>11.40<br>57.10	5.42%<br>9.19%<br>-3.45%<br>-6.88%<br>5.09%<br>-0.41%<br>8.79%	inactive		
504339	3085416	56462987	qHTS Assay for inhibitors of JMJD2A-tudor domain	0.457<br>2.286<br>11.41<br>57.07<br>113.8	-9.816%<br>-11.25%<br>-24.22%<br>-28.39%<br>-26.61%	inactive	0.01% Tween-20	
720542	3085416	56462987	qHTS for inhibitors of AMA1-RON; towards development of antimalarial drug lead: primary screen	0.464<br>2.32<br>11.61<br>58.24	-3.38%<br>-6.06%<br>-8.76%<br>-6.41%	inactive	information not provided	
624168	3085416	56462987	uHTS identification of small molecule activators of alpha dystroglycan glycosylation	10	0.296%	inactive	0.01% Tween-20	cellular assay; AlphaLISA format
504329	3085416	56462987	Discovery of small molecule probes for H1N1 influenza NS1A	12.5	9.42%	inactive	none	
623870	3085416	56462987	ARNT-TAC3: AlphaScreen HTS to detect disruption of ARNT/TAC3 interactions measured in biochemical system using plate reader - 2158-01_Inhibitor_SinglePoint_HTS_Activity	9.99	0.65%	inactive	0.02 % Tween-20	
1272365	3085416	406214639	SSB-PriA antibiotic resistant target AlphaScreen	33	-2.42%	inactive	0.01% Triton X-100	
651724	3085416	56462987	qHTS assay for inhibitors of the CtBP/E1A interaction	57.5	7.66%	inactive	0.02% Tween-20	
651725	3085416	56462987	qHTS assay for inhibitors of the Six1/Eya2 interaction	57.5	-2.63%	inactive	0.02% Tween-20	
651723<br>651687	3085416	56462987	MLPCN PGC1a modulators measured in cell-based system using plate reader - 2139-	15.56<br>15.56	-24.50%<br>-9.32%	inactive	information not provided	lytic cellular assay; lysis buffer may contain detergent

			01_Inhibitor_SinglePoint_HTS_Activity					
540317	3085416	56462987	HTS for inhibitors of HP1-beta chromodomain interactions with methylated histone tails	0.458<br>2.291<br>11.40<br>57.01<br>113.8	24.25%<br>20.82%<br>10.03%<br>0.75%<br>7.75%	inactive	0.01% Tween-20	
488953	73265241	90340581	qHTS validation assay for inhibitors of HP1-beta chromodomain interactions with methylated histone tails	0.018<br>0.091<br>0.457<br>2.290<br>11.40<br>57.10<br>114.0	-1.37%<br>-0.01%<br>15.65%<br>15.69%<br>5.59%<br>-7.87%<br>-10.82%	inactive	0.01% Tween-20	
743279	3085416	56462987	qHTS for inhibitors of inflammasome signaling: IL-1-beta AlphaLISA primary screen	11.5<br>57.5	-23.28%<br>-28.88%	inactive	none	nonlytic cellular assay; AlphaLISA format
1259354	11957587	348438277	Small-molecule inhibitors of ST2 (IL1RL1)	17	information not provided	inactive	Tween-20	AlphaLISA format
1347059	73265241	90340581	CD47-SIRPalpha protein protein interaction – Alpha assay qHTS validation	0.002<br>0.012<br>0.061<br>0.307<br>1.530<br>7.660<br>38.30	1.58%<br>1.24%<br>0.32%<br>1.49%<br>-0.63%<br>-12.51%<br>-12.42%	inactive	0.05% IGEPAL CA-630	

Supplementary Table 3. Normalized fluorescence quench rates of ivermectin analogs. Compounds were tested at 10 μM final concentrations. The results in the top eight rows were done using a blinded library; the results in the bottom two rows were controls. Data are mean ± SD for three biological replicates (independent LUV batches). Source data are provided as a Source Data file.

**compound**	**mean *Rate*/*Rate*$_{contorl}$**	**SD**
ivermectin, **1a**	6.5	1.3
ivermectin B_{1a} monosaccharide, **3**	7.7	1.3
ivermectin B_{1a} aglycone, **4**	7.4	1.5
doramectin, **8**	6.1	1.5
avermectin B_{1b}, **10**	6.8	0.3
delta2-avermectin B_{1a}, **11**	7.2	1.1
avermectin B_{1a} aglycone, **12**	3.6	0.5
remdesivir, **16**	3.4	0.4
ivermectin, **1a**	6.9	1.2
5% EtOH	5.6	1.0

Supplementary Table 4. Compound quality control and source summary.

cpd	common name	sample ID	vendor	catalog #	ELSD purity, %	UV 220 nm purity, %
**1a**	ivermectin	NCGC00094047-26	Sigma Aldrich	1354309	99.5	96.7
**1b**	ivermectin	NCGC00094047-25	Santa Cruz Biotechnology	sc-203609	98.3	94.4
**1c**	ivermectin	NCGC00094047-24	MedChemExpress	HY-15310	99.7	95.6
**2**	ivermectin B_{1b}	NCGC00843246-01	Cayman Chemicals	23824	99.8	97.1
**3**	ivermectin B_{1a} aglycone	NCGC00843248-01	Cayman Chemicals	19442	99.9	97.7
**4**	ivermectin B_{1a} monosaccharide	NCGC00843239-01	Cayman Chemicals	19443	99.7	95.1
**5**	2,3-dehydro-3,4-dihydro ivermectin	NCGC00843242-01	Cayman Chemicals	23849	99.6	96.7
**6**	28-oxo ivermectin B_{1a}	NCGC00843250-01	Toronto Research Chemicals	O856970	99.4	96.3
**7**	epi-ivermectin B_{1a}	NCGC00843241-01	Cayman Chemicals	25211	99.4	96.2
**8**	doramectin	NCGC00390529-07	Cayman Chemicals	19467	99.7	96.8
**9**	selamectin	NCGC00095066-06	Cayman Chemicals	21529	pass	pass
**10**	avermectin B_{1b}	NCGC00843247-01	Cayman Chemicals	17453	93.4	86.8
**11**	delta2-avermectin B_{1a}	NCGC00843243-01	Cayman Chemicals	25119	99.4	94.6
**12**	avermectin B_{1a} aglycone	NCGC00843245-01	Cayman Chemicals	28051	99.4	95.0
**13**	avermectin B_{1a} monosaccharide	NCGC00843244-01	Cayman Chemicals	26690	94.2	87.0
**14**	abamectin	NCGC00168290-11	Cayman Chemicals	19201	- -	89.4 (mixture) [1]
**15**	moxidectin	NCGC00163732-08	MedChemExpress	HY-B0777	- -	94.4 [1]
**16**	remdesivir		Cayman Chemicals	30354	pass	pass

[1], UV 254 nm purity

Supplementary Note 1. Antiparasitic structure-activity relationships for ivermectin and analogs. The structure-activity relationships (SAR) of ivermectin and avermectin analogs has been extensively studied in a wide variety of anthelmintic, insecticidal, and acardicidal models.[20, 21] Interpreting SAR for these compounds can be complicated given their broad spectrum of activity against helminths and arthropods. However, there is multiple lines of evidence that minor structural modifications can cause significant changes in biological activity which span several orders of magnitude in terms of compound concentration. In models of *Tetranychus urticae* (red spider mite), there is a greater than ten-fold change in activity between the disaccharide and monosaccharides ivermectins/avermectins compared to their corresponding aglycones.[20] Similar findings showing the importance of the sugar moieties have been reported in other systems.[21] When tested in a *Haemonchus contortus* larval development assay, ivermectin and avermectin analogs showed evidence of SAR, including activity enhancement with hydroxylation (vs. oxo and oxime groups) at the C-5 position, and activity enhancement with double bonds at the C-22/C-23 positions in combination with a *sec*-butyl/isopropyl substituent at the C-25 position.[22] The importance of the hydroxyl group at the C-5 position was also shown in a *Trichostrongylus colubriformis* model.[23] Related, a study focusing on the inhibitory ability of avermectin analogs on *Tetranychus cinnabarinus* (carmine spider mite) showed SAR spanning four orders of magnitude.[24] Some modifications do not appear to lead to significant changes in activity (using a *Trichostrongylus colubriformis* model), however, such as acylation at the C-4'' position.[23]

Supplementary Note 2. Ivermectin is not an effective SARS-CoV-2 antiviral in high-quality clinical trials. Several reports have shown a lack of clinical efficacy of IVM for the treatment of SARS-CoV-2 in high-quality (e.g., double-blind, randomized, placebo-controlled) clinical trials.[25, 26, 27, 28, 29, 30]

Detailed author contributions

Performed Alpha technology counter-screens: JLD. Performed analytical chemistry studies (NMR, UPLC-MS): SR. Performed *C. elegans* assays: PD. Performed cellular health assays: JLD. Performed drug-induced phospholipidosis counter-screens: ADW. Performed GPCR profiling assays: XPH, BLR. Performed membrane bilayer studies: RR. Performed PubChem analyses: JLD. Performed voltage-clamp studies on voltage-dependent sodium channels: KFH. Performed SARS-CoV-2 live virus assays: RTE. Performed SARS-CoV-2 high-content assay data analyses: TCV.

Supplementary references

1. Coussens NP*, et al.* Compound-Mediated Assay Interferences in Homogeneous Proximity Assays. In: *Assay Guidance Manual* (eds Markossian S*, et al.*). Eli Lilly & Company and the National Center for Advancing Translational Sciences (2004).

2. Wagstaff KM, Rawlinson SM, Hearps AC, Jans DA. An AlphaScreen®-based assay for high-throughput screening for specific inhibitors of nuclear import. *J Biomol Screen* **16**, 192-200 (2011).

3. Irwin J*, et al.* An aggregation advisor for ligand discovery. *J Med Chem* **58**, 7076-7087 (2015).

4. Alves VM*, et al.* SCAM Detective: accurate predictor of small, colloidally-aggregating molecules. *J Chem Inf Model*, (2020).

5. Thorne N*, et al.* Firefly Luciferase in Chemical Biology: A Compendium of Inhibitors, Mechanistic Evaluation of Chemotypes, and Suggested Use As a Reporter. *Chemistry & Biology* **19**, 1060-1072 (2012).

6. Beasley CA, Hwang T-L, Fliszar K, Abend A, McCollum DG, Reed RA. Identification of impurities in ivermectin bulk material by mass spectrometry and NMR. *Journal of Pharmaceutical and Biomedical Analysis* **41**, 1124-1134 (2006).

7. Cully DF*, et al.* Cloning of an avermectin-sensitive glutamate-gated chloride channel from Caenorhabditis elegans. *Nature* **371**, 707-711 (1994).

8. Zheng Y*, et al.* Identification of two novel Drosophila melanogaster histamine-gated chloride channel subunits expressed in the eye. *J Biol Chem* **277**, 2000-2005 (2002).

9. Nakatani Y, Furutani S, Ihara M, Matsuda K. Ivermectin modulation of pH-sensitive chloride channels in the silkworm larvae of Bombyx mori. *Pestic Biochem Physiol* **126**, 1-5 (2016).

10. Shan Q, Haddrill JL, Lynch JW. Ivermectin, an unconventional agonist of the glycine receptor chloride channel. *J Biol Chem* **276**, 12556-12564 (2001).

11. Adelsberger H, Lepier A, Dudel J. Activation of rat recombinant alpha(1)beta(2)gamma(2S) GABA(A) receptor by the insecticide ivermectin. *Eur J Pharmacol* **394**, 163-170 (2000).

12. Krause RM*, et al.* Ivermectin: a positive allosteric effector of the alpha7 neuronal nicotinic acetylcholine receptor. *Mol Pharmacol* **53**, 283-294 (1998).

13. Ahern GP, Junankar PR, Pace SM, Curtis S, Mould JA, Dulhunty AF. Effects of ivermectin and midecamycin on ryanodine receptors and the Ca2+-ATPase in sarcoplasmic reticulum of rabbit and rat skeletal muscle. *J Physiol* **514 (Pt 2)**, 313-326 (1999).

14. Pimenta PH, Silva CL, Noël F. Ivermectin is a nonselective inhibitor of mammalian P-type ATPases. *Naunyn Schmiedebergs Arch Pharmacol* **381**, 147-152 (2010).

15. Priel A, Silberberg SD. Mechanism of ivermectin facilitation of human P2X4 receptor channels. *J Gen Physiol* **123**, 281-293 (2004).

16. Kauthale RR, Dadarkar SS, Husain R, Karande VV, Gatne MM. Assessment of temperature-induced hERG channel blockade variation by drugs. *J Appl Toxicol* **35**, 799-805 (2015).

17. Chen IS, Tateyama M, Fukata Y, Uesugi M, Kubo Y. Ivermectin activates GIRK channels in a PIP(2) -dependent, G(βγ) -independent manner and an amino acid residue at the slide helix governs the activation. *J Physiol* **595**, 5895-5912 (2017).

18. Rendic SP. Metabolism and interactions of Ivermectin with human cytochrome P450 enzymes and drug transporters, possible adverse and toxic effects. *Arch Toxicol* **95**, 1535-1546 (2021).

19. Su X, Young EW, Underkofler HA, Kamp TJ, January CT, Beebe DJ. Microfluidic cell culture and its application in high-throughput drug screening: cardiotoxicity assay for hERG channels. *J Biomol Screen* **16**, 101-111 (2011).

20. Shoop WL, Mrozik H, Fisher MH. Structure and activity of avermectins and milbemycins in animal health. *Vet Parasitol* **59**, 139-156 (1995).

21. Chabala JC*, et al.* Ivermectin, a new broad-spectrum antiparasitic agent. *Journal of Medicinal Chemistry* **23**, 1134-1136 (1980).

22. Michael B, Meinke PT, Shoop W. Comparison of ivermectin, doramectin, selamectin, and eleven intermediates in a nematode larval development assay. *J Parasitol* **87**, 692-696 (2001).

23. Mrozik H, Eskola P, Fisher MH, Egerton JR, Cifelli S, Ostlind DA. Avermectin acyl derivatives with anthelmintic activity. *Journal of Medicinal Chemistry* **25**, 658-663 (1982).

24. Zhang J*, et al.* Synthesis, biological activities and structure-activity relationships for new avermectin analogues. *Eur J Med Chem* **121**, 422-432 (2016).

25. López-Medina E*, et al.* Effect of Ivermectin on Time to Resolution of Symptoms Among Adults With Mild COVID-19: A Randomized Clinical Trial. *Jama* **325**, 1426-1435 (2021).

26. Lim SCL*, et al.* Efficacy of Ivermectin Treatment on Disease Progression Among Adults With Mild to Moderate COVID-19 and Comorbidities: The I-TECH Randomized Clinical Trial. *JAMA Intern Med* **182**, 426-435 (2022).

27. Naggie S. Ivermectin for Treatment of Mild-to-Moderate COVID-19 in the Outpatient Setting: A Decentralized, Placebo-controlled, Randomized, Platform Clinical Trial. *medRxiv*, 2022.2006.2010.22276252 (2022).

28. Naggie S*, et al.* Effect of Higher-Dose Ivermectin for 6 Days vs Placebo on Time to Sustained Recovery in Outpatients With COVID-19: A Randomized Clinical Trial. *JAMA* **329**, 888-897 (2023).

29. Bramante CT*, et al.* Randomized Trial of Metformin, Ivermectin, and Fluvoxamine for Covid-19. *New England Journal of Medicine* **387**, 599-610 (2022).

30. Reis G*, et al.* Effect of Early Treatment with Ivermectin among Patients with Covid-19. *New England Journal of Medicine* **386**, 1721-1731 (2022).
