# Supplementary material for: Nonspecific membrane bilayer perturbations by ivermectin underlie SARS-CoV-2 in vitro activity
Source: bioRxiv. 2023 Oct 24:2023.10.23.563088. Preprint. [Version 1] doi: 10.1101/2023.10.23.563088 (PMC10634736; doi:10.1101/2023.10.23.563088)

File ..mschem\11-21\291121-NCGC00094047-26-03510.D Tgt Mass (EZX): 874.51
Injection Date : 29-Nov-21, 15:14:33 Seq. Line : 0
Sample Name : NCGC00094047-26 Location : D1F-A4
Acq. Operator : Zina Itkin Inj : 1
Spec. Reported : UV Integration Inj Volume : -3 ul
Acq. Method : C:\Users\Public\Documents\ChemStation\1\Methods\FINAL_GRAD_NO_PRINT.M
Analysis Method : C:\Users\Public\Documents\ChemStation\1\Methods\FINAL_GRAD_NO_PRINT.M
Sample Info : 0380650810 WalkUp method: 'FINAL_GRD_NO PRINT' Mol Wt: 874.51
Method Info : FINAL GRD BUT NO PRINT

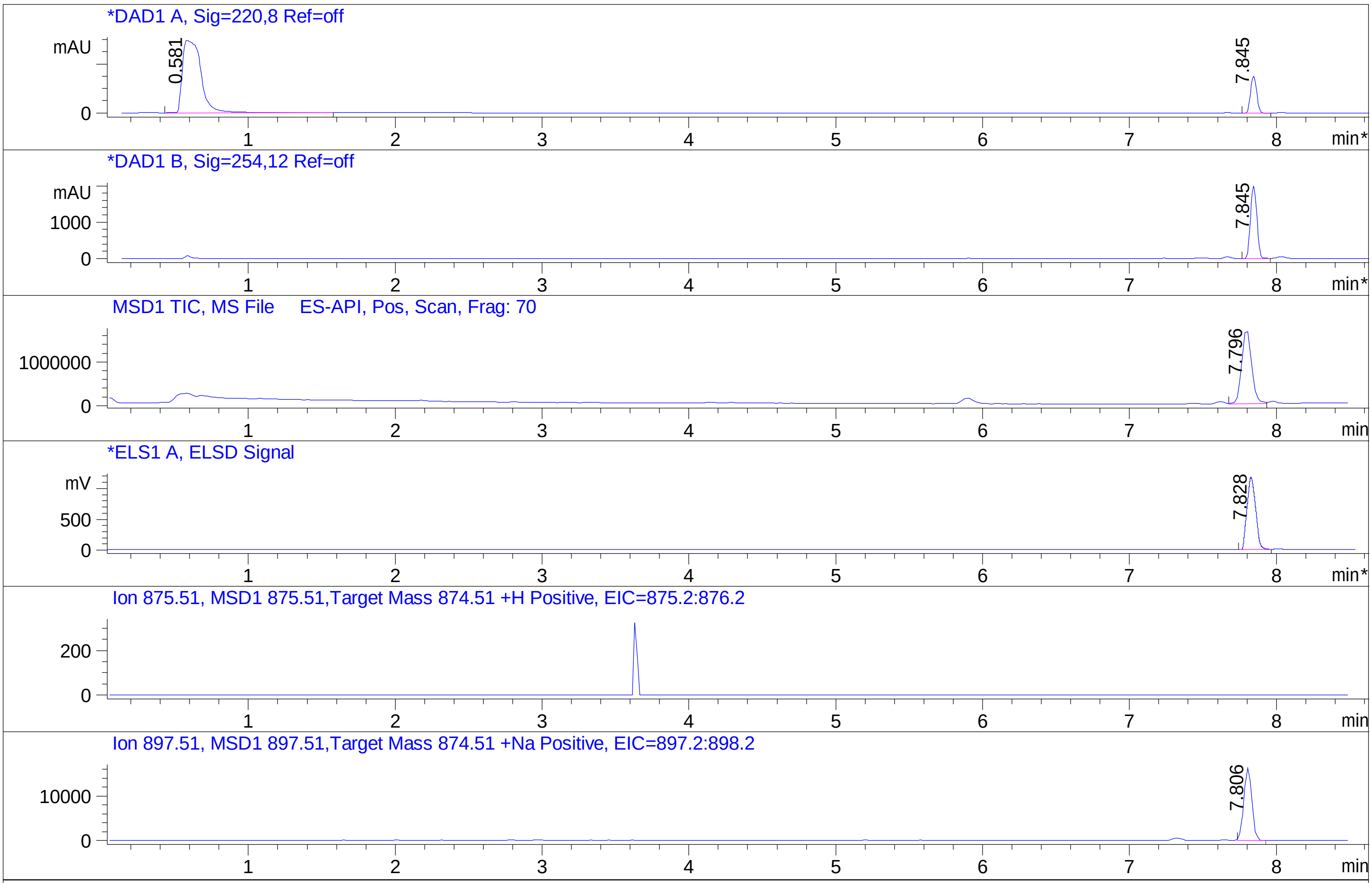

Integration Results for DAD1 A, Sig=220,8 Ref=off

RetTim	Width	Area	Height	Area%	MS(+)
0.58	0.12	25976.43	2953.10	85.49	179
7.84	0.05	4410.07	1522.47	14.51	551

Integration Results for DAD1 B, Sig=254,12 Ref=off

RetTim	Width	Area	Height	Area%	MS(+)
7.85	0.05	6051.58	2001.91	100.00	551

Ret. Time: 0.58 <<<< POSITIVE SPECTRA >>>> Ret. Time: 7.84

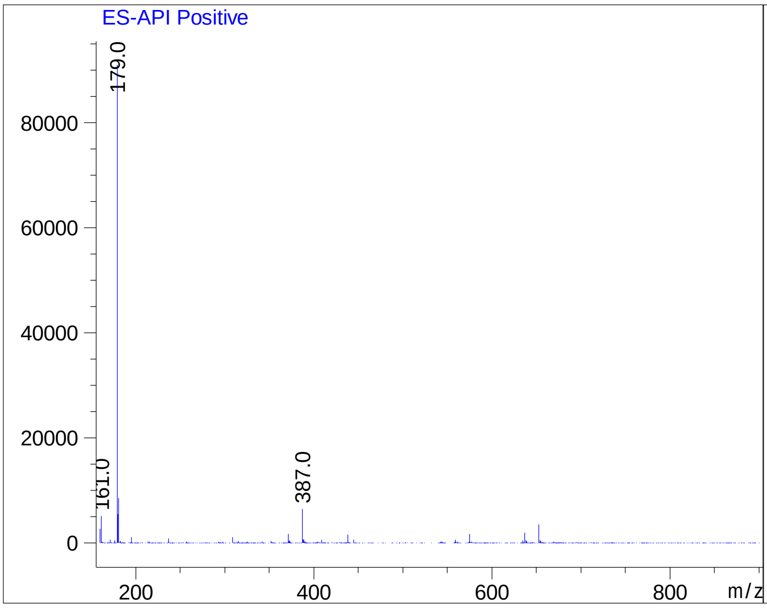

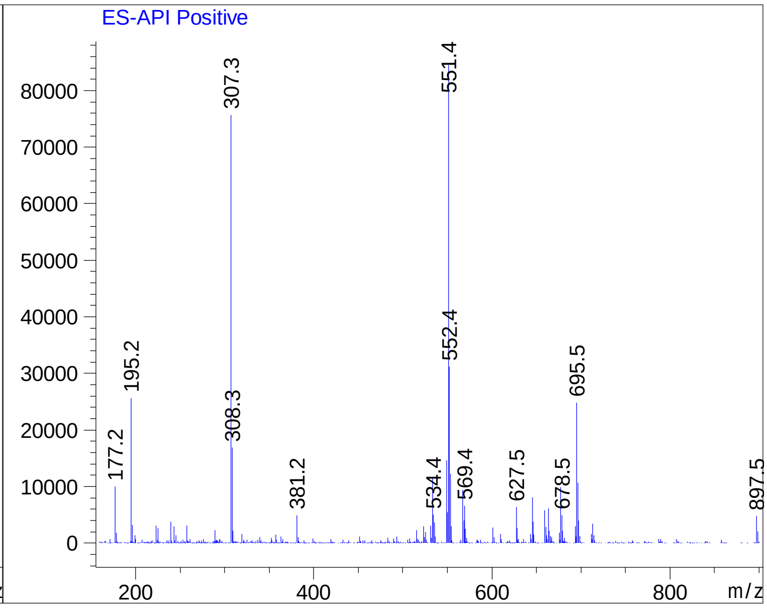

Supplement: Supplement 3 [file media-3.zip › Ivermectin-V10-SourceData2/Ivermectin-1a-UPLC-MS.D/Report.PDF]
